# Effects of a Reminiscence Program on Meaning of Life, Sense of Coherence and Coping in Older Women Living in Nursing Homes during COVID-19

**DOI:** 10.3390/healthcare10020188

**Published:** 2022-01-19

**Authors:** Alicia Sales, Sacramento Pinazo-Hernandis, Dolores Martinez

**Affiliations:** 1 Department of Development Psychology, Faculty of Psychology, University of Valencia, 46010 Valencia, Spain; 2 Department of Social Psychology, Faculty of Psychology, University of Valencia, 46010 Valencia, Spain; sacramento.pinazo@uv.es (S.P.-H.); domca24@hotmail.com (D.M.)

**Keywords:** reminiscence, meaning of life, sense of coherence, coping, older women, nursing home

## Abstract

Aging is a dynamic process that can bring well-being but also physical and cognitive decline. Older adults can draw on their personal resources to help them cope and thrive through the aging process. Having personal resources to cope and ensure older adults’ well-being is important. Psychological strengths such as a sense of coherence, resilience, and coping are protective against the adversity associated with health problems such as those stemming from the COVID-19 pandemic. Our study’s purpose was to investigate the usefulness of reminiscence therapy for older women living in nursing homes during the COVID-19 pandemic. A sample composed of 29 older women was evaluated with the Purpose-in-Life Test (PIL), Sense of Coherence (SOC-13) and Brief Cope Inventory (COPE-28). Our reminiscence program consisted of 10 sessions lasting 60 min each. Reminiscence therapy is a psychological intervention for older adults to assist in remembering and interpreting the life events, feelings, and thoughts that define and give meaning to the person’s life. Reminiscence can lead to positive mental health and other elements of particular relevance to older adults. In each session, we worked on a different theme that promoted the memory of positive emotions: optimal experience, decisive moment, stresses, tensions, problems and solutions, memories of childhood, adolescence, maturity, significant people in life, sense of life, and future script. We compared an intervention group (*n* = 12) with a control group (*n* = 17) using a pre-post, single-blind design. Significant results were obtained and showed that reminiscence therapy was effective in increasing meaning of life, sense of coherence, and coping in older women. The reminiscence therapy applied yielded positive effects in older female participants living in a nursing home during COVID-19 pandemic.

## 1. Introduction

The aging process is associated with the possible risk of adversity and loss (reduced functional capacity, illness, loss of loved ones, etc.). As such, personal resources are important to help older adults cope and thrive in later life [1]. Older women’s quality of life and well-being becomes particularly relevant given that they constitute the majority of older people and they have a greater potential to experience health-related stress or other situations of vulnerability and social exclusion [2].

Meaning in life is defined as a sense of direction and order, the reason for existence that gives meaning to personal identity and is related to the value and importance that the person gives to his or her own life. Meaning in life is considered one of the primary components of well-being and mental health, as its presence promotes growth and recovery in individuals [3,4]. Meaning in life includes two dimensions: presence of meaning in life and search for meaning in life [4]. The presence of meaning in life is positively related to life satisfaction and positive emotions [5], adaptive coping strategies [6], and positive mental health outcomes [7], and the search for meaning in life is negatively associated with depression, neuroticism and negative emotions [8], anxiety [9] and loneliness [10].

The meaning of life is also seen as a balanced understanding, related to the sense of coherence. Sense of coherence refers to one’s ability to perceive one’s life as coherent, structured, and comprehensible. It refers to one’s ability to understand a particular situation and use available resources effectively (material, psychological, and social) [11], enabling the individual to make use of adaptive coping strategies [12]. As a result, people who display high levels of sense of coherence see the world and their environment as more complete, manageable, and meaningful [13]. A sense of coherence may protect people from stress and is associated with a lower risk of health problems [11,14].

The link between sense of coherence, good health, and psychological adjustment has been extensively studied, reporting positive correlations of sense of coherence and general and psychological well-being as well as less presence of psychosomatic discomfort and depressive symptoms. Specifically, in relation to age, there seems to be a relationship with sense of coherence that indicates that the older a person is, the greater their sense of coherence [15].

The COVID-19 pandemic has been much more than a health crisis. It has become a major source of stress and anxiety for everyone. Therefore, it is of great importance for people to cope with the stressors and adapt to the changes in overall lifestyle due to the COVID-19 pandemic and to maintain their mental health [16].

Meaning in life and sense of coherence may be negatively affected during difficult times such as the COVID-19 pandemic, affecting the ability to cope with the stress. Maintaining well-being has been difficult during the COVID-19 pandemic, where social relations and meaningful activities have been curtailed, especially for those living in nursing homes.

In this regard, some authors have shown that psychological strengths such as meaning of life, resilience, and hope have statistical significance in mental health promotion during the pandemic [7]. Specifically, these authors note that meaning of life is positively associated with life satisfaction, positive affect, emotional well-being, social well-being, and psychological well-being and negatively related to negative affect, somatization, depression, and anxiety during the COVID-19 pandemic. This suggests that meaning of life plays an important role in increasing positive mental health outcomes and reducing negative mental health outcomes [17].

As indicated in [7], meaning of life is one of the most relevant aspects of coping with stressors in difficult times, and it is important to develop flexible resources such as meaning of life and purpose in life in this difficult process [18]. Reminiscence therapy, a treatment to help older adults to remember their past lives and to maintain their identities, may enhance participants’ ability to reconnect with meaning in life and sense of coherence [19].

In this line, our study aims to develop and evaluate a psychoeducational program based on reminiscence therapy, to enhance meaning of life, sense of coherence, and coping ability in older women living in nursing homes during the COVID-19 pandemic.

## 2. Materials and Methods

### 2.1. Participants

A total of 29 Spanish older women living in nursing homes participated in the study. Participants were randomized to be assigned to a control group (CG; *n* = 17) or intervention group (IG; *n* = 12), which received a psychoeducational program based on reminiscence therapy (RT). All participants completed a questionnaire before the intervention (pre-test measure) and after the intervention (post-test measure), and a questionnaire at 3 months post-intervention (follow-up measure). The IG (12 older women) was compared with the CG (17 older women) for meaning of life, sense of coherence, and coping ability. The CG was kept on a waiting list to receive the intervention once the study was completed.

The inclusion criteria for the study were that participants were admitted at the time of the study and had been confined in the nursing home, were over 65 years old, had scores for cognitive impairment above 23 on the Mini-Mental State Examination (MMSE) [20] scale, and in the case of the CI, that they attended at least 90% of the program sessions. The exclusion criteria were the presence of a serious psychiatric disorder in the medical history, the presence of sensory deficits that prevented participation, or advanced diseases with terminal criteria (renal, cardiac, respiratory failure, terminal, active neoplasms, etc.).

**Figure 1:**
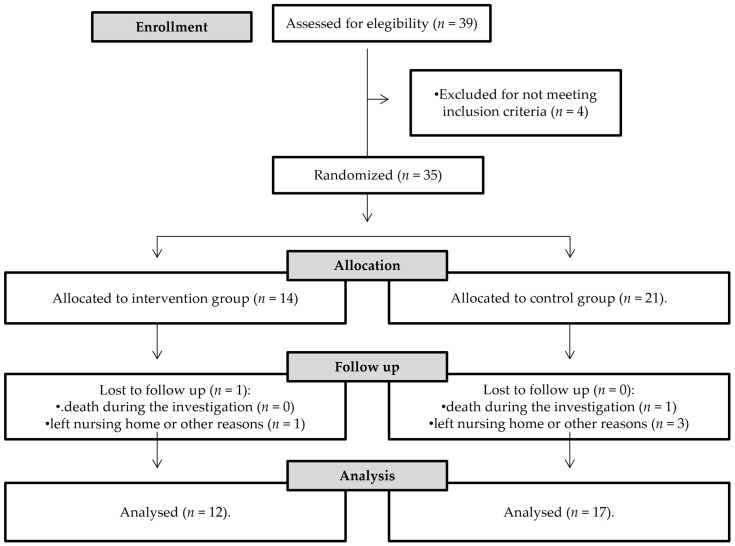
CONSORT diagram.

Of the 39 subjects, a total of 35 subjects met these criteria and signed the informed consent form. However, during the follow-up measure, 6 subjects were lost for various reasons such as leaving the residential center or death. The CONSORT diagram of the sample selection for the study is shown in Figure 1, below.

Regarding the sociodemographic variables, the mean age of the IG was 88.36 (SD = 3.77), while the mean age of the CG was 87.20 (SD = 8.05). Regarding marital status, in the IG, 92.9% were widowed and 7.1% were single, while in the CG, 75% were widowed, 10% were single, 10% were married, and 5% were separated. As for educational level, in the IG, 28.6% had no educational level, 21.4% had primary level, 28.6% had secondary level, and 21.4% had university studies; in the CG, 10% had no level, 20% had primary level, 55% had secondary level, and 15% had university studies.

The homogeneity analysis showed no differences in age (88.36 vs. 87.20; t (32) = 0.499, *p* = 0.621), marital status (Mann-Whitney z = 138.5, *p* = 0.937), and educational level (Mann-Whitney z = 117.5, *p* = 0.405). Based on these results, it can be affirmed that there were no differences between the groups in the sociodemographic variables studied.

### 2.2. Instruments

In addition to collecting sociodemographic data, various tests were administered to take pre-intervention, post-intervention, and follow-up measures. To assess life purpose, the Life Purpose Test (PIL) [21] was used, which considers the meaning of life as unique and personal for each individual and as changing throughout the life cycle. It is composed of 20 items that saturate in four dimensions: perception of meaning (motives, reasons, and valuation of living life); experience of meaning (perception of one’s own life and daily life as full of good things); goals and tasks (goals linked to concrete actions in life and perception of personal responsibility) and dialectic/destiny and freedom (tension between destiny and freedom and coping with death as an uncontrollable, unpredictable, and inevitable event). People answered on a Likert-type scale, with 7 response options and a maximum score of 140 points. A value lower than 90 points implies that the person presents existential emptiness, a value between 90 and 105 points, a lack or absence of definition with respect to the meaning of life and perceived need to discover additional meaning, and a value higher than 105 points suggests greater perceived meaning/purpose in life.

The Sense of Coherence Scale (SOC-13) [22] measures a person’s ability to perceive his or her life as coherent, structured, and comprehensible. It is composed of three key components: (1) comprehensibility (cognitive component): the degree to which people have a cognitive sense of the stimuli they encounter in the present and in the future and the ability to establish logical and time-ordered connections for what is happening in the environment and to believe that life is predictable; (2) manageability (behavioral or instrumental component): the degree to which people understand that adequate resources to cope with the demands of the environment are available to them; it is related to self-efficacy and competence; (3) meaningfulness (motivational component): the value given by the person to what happens; it refers to the person’s desire, values, emotions, and feelings that life is worthwhile.

This scale composed of 13 items consisting of statements or questions about behaviors or feelings. Respondents answer on a Likert-type scale, with 7 response options that reflect the frequency of such behaviors and feelings. The sense of coherence score ranges from 13 (low sense of coherence) to 91 points (high sense of coherence).

The Coping with Stress Scale (COPE-28) [23] is composed of 28 items that measure the coping style of a person, grouped into 14 subscales (see Table 1). In this study, and following the theory of Lazarus and Folkman [24], we divided the coping process into (1) active or adaptive coping strategies, which refers to efforts to confront the problem directly and includes activities that minimize the impact of the critical situation, emotional regulation, positive interpretation, or search for meaning (active coping, planning, positive reframing, acceptance, humor, religion, use of emotional support, use of instrumental support) and (2) passive or maladaptive coping, which refers to forms of avoidance of the situation and implies lack of commitment to the adverse situation (self-distraction, denial, venting, substance use, behavioral disengagement, and self-blame) [25]. The items are scored along a 4-point scale, with four response options as a function of frequency. The higher scores indicate a greater use of that strategy.

### 2.3. Procedure

Reminiscence focuses on re-evaluating the interpretations and emotions of past events that define and give meaning to the person’s life [26]. It is a process that promotes acceptance of self and others, seeks to accept past events, identifies a pattern of continuity, and finds meaning and value in life as it was lived, including the search for meaning in life [26].

An RT was carried out based on earlier research [27], but the sessions were adapted to the personal characteristics of the participants and objectives of the present study. We used several strategies and activities that researchers had used in the past.

The RT was conducted in a suitable room, where it was possible to maintain the physical distance of 2 m (6 feet), in a nursing home in October 2020. The program was highly appreciated by the participant. The pretest measure was taken in July 2020, and the post-test measure in October 2020. Finally, the follow-up measure was taken in January 2021, coinciding with the fourth wave of the COVID-19 pandemic; many people were affected by SARS-CoV-2 in the center, locked down in their rooms and isolated during most of the day.

The ultimate goal of the RT was to enhance the empowerment of the participants and to conduct an appropriate life review that would allow for the development of wholeness. Most people have many natural coping mechanisms to cope with the various demands of daily life, but in very stressful times with uncertainty and no control, such as the time lived during the confinement produced by the pandemic, and in people with depression or anxiety disorder, and even more so at the end of life, these circumstances could overwhelm coping capacities, and people may not be able to use their existing behavioral repertoires.

For participants to be able to cope as optimally as possible, RT should develop improved coping and a basic understanding of life circumstances. Beginning with the individualized experienced-based knowledge of the participants, basic ideas about a life review were developed. Individual opinions were appreciated and respected. The empowerment of the participants was also a key element in the program. Many take-home tips were provided. The list of topics covered in each session was very useful to bring out emotions and associated feelings that could be discussed as a group.

#### Reminiscence Therapy Program

This program consisted of one introductory session and ten intervention sessions, carried out over six weeks (two sessions per week), of approximately 90 min each and performed by a trained and expert psychologist. In addition, it was executed in a group setting composed of 12 participants (IG) [26].

Ten sessions were conducted with all the participants and the psychologist. Each session focused on a topic: optimal experience in life (session 1); lowest moment in life (session 2); decisive moment (session 3); earliest memory (session 4); memories of adolescence (session 5); memories of adulthood; (session 6); significant people (session 7); sense of life and future script (session 8); stressors and solutions (session 9); ideology, experiences, and values (session 10).

At the presentation and topic introduction, the psychologist introduced general reflections on the topic. For example, at the first session, optimal experience, happiness was described, including different and individual experiences of happiness along life were. At the second and third sessions, lowest and highest moments were shared, based on a documentary in which a well-known public figure shared that life is made up of small moments to enjoy and appreciate. At the fourth, fifth and sixth sessions (childhood, adolescent, and adult memories), songs, important dates in the life of each person, and historical events were used to help people remember parts of their lives. The seventh session (significant people) revolved around the significant people in one’s life and the exercise of gratitude, with the excuse of writing them a letter. The eighth session (meaning of life) was intended to help participants think about the meaning and significance of their lives. Each participant had to write her future plan, thinking about what she would like to do and learn, and with whom. The nineth session (stressors and solutions) began by encouraging individuals to think about problems that generated stress, looking for the cause of the problem and possible solutions, which were discussed by all. The last session (beliefs and values) allowed participants to share ideologies and values, including thoughts linked to spirituality and religiosity, sense of belonging, immigration and social problems, and what can we do as individuals and as a group.

After each individual exercise, there was a sharing of the group’s reflections. Some comments that were shared by the participants were “Life has given me problems, but it has not taken away my smile”; “In life you have to be patient because everything passes and everything comes”; “Life’s problems have made me stronger;” “Never give up”.

Group activities and experiences, such as relaxation, stress breathing techniques, smells, humor and humorous laughter, painting, writing, dancing, and singing songs known to the participants and fun songs such as Macarena or I Will Survive were included. Nearly all were strongly linked to memories and included different senses (sight, smell, hearing, etc.) that helped evoke memory.

Final conclusions and proposals were shared with the participants in each session, with homework for the next session, including reflections, reading a poem, writing some thoughts and dreams, thinking about parents, brothers and sisters, school teachers, childhood, adolescence, or marriage, searching for pictures to share in the next session, and thinking of time shared with loved ones and moments of joy, thinking of favorite foods and places where you ate them or people who cooked them well.

All sessions, with the exception of the introduction session, were divided into the following parts: welcome to the group, presentation and topic introduction (15 min), general reflections on the topic (20 min), individual exercise (10 min), sharing of exercises and reflections (15 min), group dynamics (15 min), final conclusions, proposals for the next session (10 min), and farewell (5 min). Table 2 below shows the objectives addressed in each session.

### 2.4. Data Analysis

We performed *t*-test analysis, repeated measures analysis, and analysis of variance. All analyses were carried out using the SPSS 26 statistical package (IBM, New York, NY, USA). The F-test was used to compare the two standard deviations of two samples, and η^2^ was used as a measure of effect size; it reflects the percentage of the variance in a dependent variable explained by the independent variables in a sample.

We performed *t*-tests for independent samples and chi-squared tests to determine whether the groups were homogenous prior to treatment. To analyze the intervention’s effects, repeated measures analysis of variance was conducted for the three main dimensions (1. Meaning in Life—PIL; 2. Sense of Coherence—SOC-13; 3. Coping with Stress Strategies—COPE-28), applying the Bonferroni correction (α < 0.05). Sample effects as well as interaction effects (group x time) were examined for all sub-dimensioned (four dimensions in meaning in life: (a) meaning perception, (b) meaning experience, (c) goal and tasks, and (d) fate–freedom dialectic; three dimensions in sense of coherence: (a) comprehensibility, (b) manageability and (c) significance; and two dimensions in coping with stress strategies: (a) active coping, (b) passive coping). The level of statistical significance employed was *p* < 0.05.

## 3. Results

The RT showed a significant impact across all dimensions of meaning in life: meaning perception (F2, 54 = 776.01; *p* = 0.000; η^2^ = 0.669); meaning experience (F2, 54 = 56.46; *p* = 0.000; η^2^ = 0.667); goals and tasks (F2, 54 = 54.97; *p* = 0.000; η^2^ = 0.614); and fate–freedom dialectic (F2, 54 = 62.73; *p* = 0.000; η^2^ = 0.699).

Comparisons of the groups at the different time points are presented in Table 3 below. Comparisons of the evolution of the groups over time are presented in Table 4. Significant differences were observed in the IG throughout the three times in the four scales. In all cases, a trend of significant improvement is observed from the pre-test to the post-test score. However, from the post-test to the follow-up measure, a significant regression is observed, returning to the initial levels (see means in Table 3). In the CG case, a significant decreasing trend is observed at all times, both from pre-test to post-test and from post-test to follow-up measures (see means in Table 3).

Secondly, the sense of coherence showed a significant effect for the time-group interaction in the dimensions of comprehensibility (F2, 54 = 8.95; *p* = 0.003; η^2^= 0.249) and significance (F2, 54 = 8.46; *p* = 0.004; η^2^= 0.239), but not for the dimension of manageability (F2, 54 = 2.39; *p* = 0.131; η^2^= 0.082).

Table 5 below shows the comparisons of the groups at the different time points. Table 6 shows the comparisons of the evolution of the groups over time. In the IG, the maintenance of scores from pre-test to post-test was observed in all scales (see means in Table 6), and a significant decrease in comprehensibility and significance scales in the measures from post-test to follow-up was also seen. In the CG, a significant decreasing trend at all times, both from pre-test to post-test and from post-test to follow-up, was measured in all scales (see means in Table 6), excluding the post-test measure to follow-up measure on the significance scale.

Finally, regarding the Coping with Stress Scale (COPE-28), we observed that the RT had a significant effect on both active coping (F2, 54 = 23.45; *p* = 0.000; η^2^ = 0.474) and passive coping (F2, 54 = 20.21; *p* = 0.000; η^2^ = 0.437). When analyzing the simple effects in active coping, it was observed that there were no significant differences between groups in the scores of the pre-test and post-test measures, but there were in the follow-up measures; while in passive coping, significant differences were observed in the scores of the pre-test and follow-up measures, but not in the post-test measures (see Table 7).

## 4. Discussion

RT has been shown to be effective in promoting mental health and well-being during aging, as well as in coping with major life events and finding meaning in life [28]. In the present study, we analyzed the efficacy of RT performed after the situation provoked by the COVID-19 pandemic, obtaining positive results for meaning of life and sense of coherence as well as for coping strategies in a sample of older women living in nursing homes.

In relation to the meaning of life, RT in older women showed a significant effect on the time-group interaction in all its dimensions: perception of meaning, experience of meaning, goals and tasks, and the destiny–freedom dialectic. The participants showed more sense of life by having motives and reasons to live after the RT; they made a more positive evaluation of life; they perceived their own life and daily life with meaning, full of good things; they had goals linked to concrete actions in life; they had a greater perception of personal responsibility and capacity to decide their destiny.

There are few studies that have analyzed the effect of RT on the meaning of life [29,30,31]; however, it should be noted that all obtained higher scores after the intervention, which supports our results, suggesting that an intervention in reminiscence is an effective tool to improve the meaning of life of older women living in nursing homes, and much more so during the COVID-19 pandemic. Taking into account the results of RT, and comparing the changes over time of the IG with the CG, we can affirm that the RT performed enhances people’s ability to elaborate on and celebrate meaning in life. Different studies have shown that lack of meaning of life is associated with increased risk of mortality. On the contrary, the presence of meaning of life is associated with subjective well-being and positive feeling of life and life satisfaction [32], factors that predict longevity and healthy aging. People who feel satisfied with their life integrate the good and bad moments of their life trajectory and perceive their life in a coherent way; that is, they have the ability to understand how their life is organized and how they place themselves in front of the world, giving value to what happens. They think that life is worthwhile, are organized, and are able to give coherence to their life. Some authors [33] find positive correlations between the sense of coherence and the meaning of life. Thus, there is a relationship between the subject’s ability to understand how his or her life is organized and how it is positioned in the world, the ability to manage it and to feel that it has meaning, and furthermore, that life is oriented towards goals that the subject wishes to achieve. In relation to the sense of coherence, a significant effect for the time-group interaction in the comprehensibility and meaningfulness dimensions was observed but not for the manageability dimension. Comprehensibility is the capacity of people to understand how their life is organized and how they face the world. Clearly the review of life moments and the reinterpretation of them may have helped to increase the understanding of their life as a whole and the meaning and significance of it.

The participants, after the RT, presented more comprehensibility but less manageability and also less significance, thus subtracting value from life and thinking that life is not worth living. These results make even more sense and have more impact if we analyze them in the context of the COVID-19 pandemic; the participants know and are aware of the situation we live in but do not have the resources to solve it. In addition, after having suffered losses, uncertainty, fear, insecurity, and loneliness, they feel that they have already fulfilled their mission in life, showing no interest in continuing to live. However, we do not know of any research on RT that has studied the sense of coherence to which we can compare our results, nor of any in the context of the COVID-19 pandemic. With respect to the CG, there is a decrease in the score in sense of coherence in all dimensions and times. This variable must be taken into account in order to promote change by professionals in nursing homes due to its impact on health and psychological adjustment [34]. Older adults who achieve a high sense of coherence are able to make sense of and accept their life story. They are able to manage their life history and accept their past, achieving wholeness [35]. When people can give meaning of life and sense of coherence to their life, this allows and facilitates the resolution of problems in an adaptive way and above all in stressful situations [33].

The coping strategies applied to resolve internal and external conflicts may favor successful aging, as suggested by different studies that analyze different types of coping used by older adults and their effects and relate them to psychological health [36]. Regarding coping in our study, a significant effect of the RT was observed for the time-group interaction in both active coping and passive coping. The participants after the RT presented more active coping; they confronted problems directly by performing activities that minimized the impact of the critical situation, regulated emotions, interpreted positively and searched for meaning. They presented less passive coping: avoiding the situation and not engaging in resolving the adverse situation. Our results support other studies [37,38,39,40], which indicates that older adults use active coping strategies to a greater extent; they initiate direct actions, increase their own efforts to eliminate or reduce the stressor, and plan strategies and direction of action to cope with the stressful situation. In short, they use functional strategies by presenting an active and direct coping style, reevaluating in a positive way, and taking measures of social support and emotional expression.

The effects of the intervention were maintained at follow-up in active coping, which shows that RT provided resources (positive reframing, emotional and instrumental support) to the older women in the research to cope with adverse situations. This is especially appropriate for the challenges of the pandemic. However, there was an increase in passive coping, which could be due to non-acceptance of the COVID-19 pandemic situation and a lack of control (manageability) to improve it.

With respect to the CG and in the follow-up measure, we found a very significant decrease in active coping and increase in passive coping; when compared to the IG, we can affirm that the RT improved coping skills in older women, results supported by different research [7,41,42,43]. Some results [41] found that reminiscence therapy increased the social contact between participants and promoted the importance of social support in coping with changes and losses, encouraging people to ask for help and seek social support. On the other hand, other results [42] found that adaptation to old age increased after the application of an 8-week individual reminiscence therapy to older women.

## 5. Conclusions

RT allows older women to reorganize, reinterpret, and integrate the events of the past, perceiving their life as more organized and coherent. This permits them to understand how their life is organized and how they situate themselves in front of the world, which enables and/or allows them to face problems and resolve situations in a more positive way, including in isolation due to the confinement established by the COVID-19 pandemic. Having certain psychological strengths, such as a sense of life, a sense of coherence, coping strategies, and resilience, enables individuals to cope with stressors, adapt to changes due to COVID-19, and maintain their well-being and mental health. This is the reason why RT can significantly benefit the quality of life of the older adults living in nursing homes during lockdowns and isolation situations.

One of the main reasons for the loneliness felt during the COVID-19 pandemic has been a lack of companionship [43]. Loneliness is a predictor of depression [44,45,46] and one of the primary reasons for suicidal ideation [47]. Based on data from different surveys [48], depression has increased during the COVID-19 pandemic, and even more so in countries with high death rates and stringent measures and in older adults living alone or who are lonely.

Preventative measures for increasing mental well-being among those at-risk groups are needed to minimize the negative consequences of the COVID-19 pandemic. Interventions could focus on increasing the personal capacity to improve one’s resilience.

RT was performed in a group setting, which produced added benefits, because feeling like part of a group and receiving social support moderates loneliness levels [49].

In addition, this improvement could reduce the generalized consumption of psychotropic drugs, which has increased in such centers during the COVID-19 pandemic, reducing the overload and burnout of professionals and caregivers.

## 6. Limitations

Finally, some limitations of this study should be noted. The first and most important is the sample loss and the reduction of the number of participants. The results are very positive; however, if they could be replicated with a larger sample, it would increase their power. The second limitation is the time when the third measurement (follow-up measurement) was taken, since the pandemic had not ended, and the time coincided with a new wave (the fourth wave of the pandemic) and many positive cases in the centers and thus with a return to confinement in their rooms (and isolation). Given that it seems that the variables measured in our study improved after the program, it would be recommended to replicate the study in larger samples using both institutionalized and non-institutionalized participants and 3-month and 6-month follow-up measures. The third limitation is that well-being was not measured directly. In future studies, it could be interesting to measure well-being.

## Figures and Tables

**Table 1:** Subscales of the Coping with Stress Scale (COPE-28).

Subscale	Definition
Self-distraction	Concentrate on other projects, trying to distract oneself with other activities to try not to focus on the stressor
Active Coping	Initiating direct actions, increasing one’s own efforts to eliminate or reduce the stressor
Denial	Denying the reality of the stressful event
Emotional Support	Receiving emotional support, such as sympathy and understanding from other people
Social and Instrumental Support	Seeking help, advice, or information from competent people about what to do
Behavioral Disconnection	Reduce efforts to cope with the stressor; may give up and no longer strive to achieve the goals in which the stressor interferes
Emotional Relief	Tendency to express or vent feelings and emotions caused by the stressor, producing an increased awareness of one’s own emotional discomfort
Positive Reinterpretation	Look for the positive and favorable side of the problem and try to improve or grow personally from the situation
Planification	Plan strategies and direction of action to cope with the stressful situation
Humor	Make jokes or mock the stressor and laugh at the situation
Acceptance	Accepting the reality of the fact that it is happening
Religion	Increasing participation in religious activities in stressful situations
Self-incrimination	Blaming oneself for what happened
Substance abuse	Use of alcohol or other substances in order to feel good or to help you cope with the stressor

**Table 2:** Objectives of sessions in the reminiscence program.

Session	Objectives
1. Optimal Experience	Recover autobiographical memories of positive events, reinterpret positive experiences and emotions, consolidate the identity and integrity of the self by evaluating achievements and giving meaning to life.
2. Lowest Moment	Recover autobiographical memories of negative events, reinterpret negative experiences and emotions, consolidate identity and integrity of self by evaluating coping strategies.
3. Decisive Moment	Recovering specific autobiographical memories and “decisive moments of life”, reconstructing and searching for meaning and sense of past events, consolidating identity, coherence, and attribution of meaning to experiences.
4. Earliest memory	Recover autobiographical memories and construction of life episodes from childhood, evoke positive or negative emotions and integrate them into the present to consolidate meaning and coherence for experiences.
5. Memories of adolescence	Reconstructing past events of adolescence and searching for meaning, consolidating identity and integrity of self, coherence and attribution of meaning to experiences.
6. Memories of adulthood	Recover autobiographical memories of positive and negative events of adulthood, consolidate the identity and integrity of the self by evaluating achievements and failures and giving meaning and coherence to life.
7. Significant people	Reconstructing events with important and significant people in our lives, reconstructing these events and searching for meaning, incorporating aspects of intervention based on gratitude, forgiveness, and emotions, reinforcing social bonds.
8. Sense of life and future script	Recovering memories of positive and negative events of the past and integrating them into the present to consolidate integrity, coherence, and the attribution of meaning to the experiences of the self, giving meaning to life, fostering a positive attitude and maintaining the feeling of continuous development and improvement over time.
9. Stressors and solutions	Reconstructing and working on negative events of the past, normalizing negative moments, working on and improving coping and adaptation strategies.
10. Ideology: experiences and values	Give value to beliefs and values giving meaning to life in order to consolidate identity, integrity, coherence, and meaning attribution to the experiences of the self.

**Table 3:** Mean meaning of life dimensions at the three time measures and univariate comparison statistics in the IG and CG.

	Time	IG	CG	F	*p*	η^2^
Meaning perception	Pre-test	44.08	46.17	0.375	0.546	0.014
	Post-test	49.83	39.70	13.36	0.001	0.331
	Follow-up	44.91	30.76	33.78	0.000	0.556
Meaning experience	Pre-test	31.16	32.88	0.321	0.575	0.012
	Post-test	37.50	27.29	14.39	0.001	0.348
	Follow-up	32.83	19.29	37.46	0.000	0.581
Goals and tasks	Pre-test	33.33	34.64	0.247	0.623	0.009
	Post-test	39.08	29.64	17.10	0.000	0.388
	Follow-up	34.75	22.82	46.44	0.000	0.632
Fate–freedom dialectic	Pre-test	16.66	17.05	0.170	0.683	0.006
	Post-test	17.91	14.70	12.35	0.002	0.314
	Follow-up	16.58	10.47	60.57	0.000	0.692

**Table 4:** Univariate analysis of the evolution of the groups independently over time.

		F	*p*	η^2^	Time	*p*
Sense perception	IG	40.63	0.000	758	Pre-post	0.000
					Post-Follow-up	0.000
	CG	97.03	0.000	0.882	Pre-post	0.000
					Post-Follow-up	0.000
Sense experience	IG	50.45	0.000	0.795	Pre-post	0.000
					Post-Follow-up	0.000
	CG	62.16	0.000	0.827	Pre-post	0.000
					Post-Follow-up	0.000
Goals and tasks	IG	68.49	0.000	840	Pre-post	0.000
					Post-Follow-up	0.000
	CG	53.40	0.000	0.804	Pre-post	0.000
					Post-Follow-up	0.000
Fate–freedom dialectic	IG	7.54	0.003	0.367	Pre-post	0.004
					Post-Follow-up	0.028
	CG	122.7	0.000	0.904	Pre-post	0.000
					Post-Follow-up	0.000

**Table 5:** Mean of the sense of coherence dimensions at the three time points and univariate comparison statistics in experimental and control groups.

	Time	IG	CG	F	*p*	η^2^
Comprehensibility	Pre-test	26.16	27.70	0.491	0.490	0.018
	Post-test	26.41	23.58	3.13	0.088	0.104
	Follow-up	25.00	20.23	15.02	0.001	0.357
Manageability	Pre-test	20.41	22.29	1.30	0.264	0.46
	Post-test	18.00	16.82	2.64	0.116	0.089
	Follow-up	17.83	16.35	4.02	0.055	0.130
Significance	Pre-test	19.16	20.29	0.624	0.437	0.023
	Post-test	17.50	14.05	22.86	0.000	0.459
	Follow-up	16.00	13.29	14.55	0.001	0.350

**Table 6:** Univariate analysis of the evolution of the groups independently over time.

		F	*p*	η^2^	Time	*p*
Comprehensibility	IG	3.70	0.038	0.222	Pre-post	1.00
					Post-Follow-up	0.030
	CG	35.94	0.000	0.734	Pre-post	0.003
					Post-Follow-up	0.000
Manageability	IG	1.35	0.275	0.095	Pre-post	0.398
					Post-Follow-up	1.00
	CG	10.85	0.000	0.455	Pre-post	0.001
					Post-Follow-up	0.14
Significance	IG	9.00	0.001	0.409	Pre-post	0.616
					Post-Follow-up	0.001
	CG	25.45	0.000	0.662	Pre-post	0.000
					Post-Follow-up	0.138

**Table 7:** Mean active and passive coping at the three times measures and statistics.

	Time	IG	CG	F	*p*	η^2^
Active Coping	Pre-test	13.50	14.03	0.177	0.677	0.007
	Post-test	23.33	22.81	0.043	0.838	0.002
	Follow-up	24.33	11.31	0.9874	0.000	0.792
Passive Coping	Pre-test	29.83	19.00	28.36	0.000	0.522
	Post-test	13.50	12.06	0.761	0.391	0.028
	Follow-up	16.16	19.43	5.48	0.000	0.174

Significant differences were obtained in active coping both in the IG (F2, 26 = 28.01; *p* = 0.000; η^2^ = 0.691) and in the CG (F2, 26 = 51.34; *p* = 0.000; η^2^ = 0.804). In the IG, a significant increase in score was observed between the pre-test and post-test measure (*p* = 0.000), while in the CG, a significant increase was observed in both the pre-test and post-test mean (*p* = 0.000), and a decrease between the post-test and follow-up measure (*p* = 0.000). In passive coping, significant differences were also observed both in the IG (F2, 26 = 33.15; *p* = 0.000; η^2^ = 0.726) and in the CG (F2, 26 = 33.53; *p* = 0.000; η^2^ = 0.728). A significant decrease in the pre-test to post-test mean was observed in both the IG and CG (*p* = 0.000 and *p* = 0.000, respectively), and a significant increase in the post-test mean at follow-up measures in both groups (*p* = 0.051 (IG); *p* = 0.000 (CG)).

## Data Availability

Data are available upon request.
